# Periodontopathogens *Porphyromonas gingivalis* and *Fusobacterium nucleatum* and Their Roles in the Progression of Respiratory Diseases

**DOI:** 10.3390/pathogens12091110

**Published:** 2023-08-30

**Authors:** Tao Shi, Jiale Wang, Jiajia Dong, Pingyue Hu, Qiang Guo

**Affiliations:** 1 State Key Laboratory of Oral Diseases, National Clinical Research Center for Oral Diseases, West China Hospital of Stomatology, Sichuan University, Chengdu 610041, China; 2 Department of Pulmonary and Critical Care Medicine, West China Hospital, Sichuan University, Chengdu 610041, China; 3 State Key Laboratory of Respiratory Health and Multimorbidity, West China Hospital, Sichuan University, Chengdu 610041, China

**Keywords:** oral microbiota, systemic disease, periodontal disease, respiratory disease, *Porphyromonas gingivalis*, *Fusobacterium nucleatum*, microbial interaction

## Abstract

The intricate interplay between oral microbiota and the human host extends beyond the confines of the oral cavity, profoundly impacting the general health status. Both periodontal diseases and respiratory diseases show high prevalence worldwide and have a marked influence on the quality of life for the patients. Accumulating studies are establishing a compelling association between periodontal diseases and respiratory diseases. Here, in this review, we specifically focus on the key periodontal pathogenic bacteria *Porphyromonas gingivalis* and *Fusobacterium nucleatum* and dissect their roles in the onset and course of respiratory diseases, mainly pneumonia, chronic obstructive pulmonary disease, lung cancer, and asthma. The mechanistic underpinnings and molecular processes on how *P. gingivalis* and *F. nucleatum* contribute to the progression of related respiratory diseases are further summarized and analyzed, including: induction of mucus hypersecretion and chronic airway inflammation; cytotoxic effects to disrupt the morphology and function of respiratory epithelial cells; synergistic pathogenic effects with respiratory pathogens like *Streptococcus pneumoniae* and *Pseudomonas aeruginosa*. By delving into the complex relationship to periodontal diseases and periodontopathogens, this review helps unearth novel insights into the etiopathogenesis of respiratory diseases and inspires the development of potential therapeutic avenues and preventive strategies.

## 1. Oral Microbiota and Systemic Diseases

As the second largest human microbial community, the oral cavity harbors more than 700 kinds of microbes, including bacteria, fungi, protozoa, mycoplasmas, and viruses [1,2]. The colonization of oral microbiota starts during birth [3]. Moreover, the unique physiological conditions of the oral cavity provide a favorable environment for microorganisms to grow and reproduce [4,5]. As humans grow, the host interacts with oral microbiota, gradually forming stable oral biofilms called dental plaques that attach to the tooth surface and consist of oral microbes and a surrounding extracellular matrix [6]. Under normal conditions, dental plaque biofilms contribute to stimulation of the immune system and maintain oral health locally. However, the dysbiosis or imbalance of oral biofilms caused by internal or external factors can give rise to oral diseases such as dental caries, gingivitis, and periodontitis [7]. Research has increasingly suggested that oral microbial imbalance not only contributes to the above-mentioned oral diseases but is also associated with the occurrence and development of many systemic diseases, such as respiratory diseases [8,9,10,11,12], digestive system diseases [12,13,14,15], cardiovascular disease [16], rheumatoid arthritis [17], Alzheimer’s disease [18], preterm birth [19], and diabetes mellitus [20].

Currently, researchers have proposed different pathways for oral microbiota to reach other organs. The most feasible and plausible mechanism is that oral microbes and inflammatory factors locally produced in periodontal tissues can translocate into systemic circulation and spread throughout the body with the blood [21,22]. Additionally, as specific to different diseases, there may be different mechanisms for oral microbes to disseminate to extra-oral sites due to disparity in the physiological anatomy of body organs. Among respiratory diseases, periodontopathogens may arrive within the respiratory tract and lungs via the aspiration of saliva [23]. Apart from the above, oral pathogenic bacteria can reach the gastrointestinal tract during swallowing, which may be related to intestinal microbial disorders, intestinal-mediated systemic inflammatory response, and the occurrence of colorectal cancer [20,24]. Phoebus et al. [25] found that certain oral bacteria can cross the placental barrier into the amniotic fluid and fetal circulation, and this may elevate the risk of preterm birth in pregnant women. A recent study has identified *Porphyromonas gingivalis*, one of several hundred oral bacterial species, in the brain of patients with Alzheimer’s disease, indicating that periodontal microorganisms can access the brain through the blood–brain barrier [26].

Under the influence of factors such as poor oral hygiene, there is a gradual and dramatic shift in the composition of the oral microbial community originally in a symbiotic state with the host [7]. The dysbiotic oral microbial communities can induce periodontal tissues to produce a variety of proinflammatory cytokines, such as tumor necrosis factor alpha (TNF-α), interleukin-1 alpha (IL-1 α), interleukin-1 beta (IL-1 β), and interleukin-6 (IL-6) [27]. These proinflammatory cytokines disseminate through the hematologic system to reach other sites in the body, potentially exacerbating pre-existing systemic inflammation [28]. The low concentrations of bacteria lipopolysaccharide (LPS) may cause a systemic inflammatory response, leading to intravascular coagulation abnormalities and organ dysfunction [29]. Furthermore, some Gram-negative bacteria such as *P. gingivalis* provide complex long-range delivery systems for LPS and other toxins by secreting outer membrane vesicles (OMVs) [30].

## 2. Periodontal Diseases and Respiratory Diseases

Periodontal diseases are chronic inflammatory diseases that result from the dysbiosis of oral microbiota and the disorder of host inflammatory response, characterized by the resorption of alveolar bone and the destruction of periodontal tissues [31]. Diverse groups of microbes in the oral cavity play differential functional roles in the pathogenesis of periodontal diseases in different individuals, but it has been determined that some bacteria have a critical role in the pathogenesis of periodontal diseases, such as *P. gingivalis*, *Fusobacterium nucleatum*, etc. [32]. These periodontal pathogenic bacteria can induce the production of pro-inflammatory cytokines in the gums and bone tissues, causing a chronic immune inflammatory response that leads to the destruction of the structural components of periodontal tissues and eventual manifestations of gingivitis and periodontitis [31].

*P. gingivalis* is a Gram-negative anaerobic bacterium that mainly colonizes the periodontal pockets. As a member of the red complex, *P. gingivalis* is strongly associated with the development and progression of periodontal diseases [33]. Its pathogenic capacity mainly results from the following aspects: (1) Adhesion and invasion abilities mediated by fimbriae, hemagglutinins, and proteins [34]; (2) Induction of peripheral CD4+ T helper cells to produce proinflammatory cytokines, such as IL-1 and IL-6 [35]; (3) Activation of T lymphocyte immune response and promotion of receptor activator of nuclear factor-κB (NF-κB) ligand (RANKL)-induced osteoclast activation [36]; (4) Attenuation of dendritic cell-induced chemokine responses [37]; (5) Production and secretion of gingipains such as arginine- and lysine-specific cysteine proteases [38]; (6) Secretion of OMVs to deliver virulence factors [39].

*F. nucleatum* is a Gram-negative anaerobe widely found in the oral cavity of both healthy and diseased individuals. *F. nucleatum* possesses multiple adhesins that allow it to co-aggregate with almost all periodontal pathogenic bacteria, thus playing a profoundly important role in the transition of the oral microbial community from a healthy to a diseased state [40,41]. The adhesins RadD, Fap2, and FomA are major outer membrane proteins for *F. nucleatum*-mediated interspecies interactions [42,43,44], while Fusobacterium adhesion A (FadA), the best-characterized virulence factor of *F. nucleatum*, mediates *F. nucleatum* adhesion to and invasion of host cells [45]. Furthermore, *Fusobacterium nucleatum*-associated β-defensin inducer (FAD-I) can bind to toll-like receptors on gingival epithelial cells, which induces β-defension-2 production [46,47]. Similarly, RadD and Fap2 can stimulate the secretion of cytokines such as IL-6, IL-8, and TNF-α [48].

Respiratory diseases are among the leading causes of both morbidity and mortality across the world, and their lesions mainly occur in the trachea, bronchial tubes, lungs, and chest cavity [49]. Lower respiratory tract infections, chronic obstructive pulmonary disease (COPD), and lung cancer are all very prevalent respiratory diseases. Pneumonia, the leading disease of lower respiratory tract infections, is an inflammatory disorder of the lung frequently caused by bacterial or viral infections [50]. In 2019, a total of 489 million incident cases of lower respiratory infection occurs globally, and 2.5 million people die due to pneumonia [51]. The sudden outbreak of COVID-19 in recent years has attracted even more extensive global attention and research. COPD, as one of the main causes of morbidity and mortality worldwide, accounts for 49.1 deaths per 100,000 [52,53]. Lung cancer is cancer leading to the highest mortality rate, with 18 deaths per 100,000 population as a result [54].

In terms of anatomical relationship, the oral cavity and the lower respiratory tract are continuous, making the oral cavity a major source of microbiota in the lung [55]. The sophisticated and efficient immune and mechanical defense mechanisms of the airways prevent microbes from colonizing the lower respiratory tract so that the lung flora of healthy individuals is only partially identical to the oral flora with lower bacterial concentrations and less diversity [56]. However, when the host defense system is impaired to promptly clear microbes entering the lungs, oral microbes colonize the lungs and affect lower respiratory and lung diseases [57]. Alternatively, oral microorganisms may enter the lungs and affect respiratory disease when large inoculums of microorganisms exceed the clearance capacity of the defense system [58]. Accumulating studies have indicated that periodontal diseases are associated with respiratory diseases. Through a comparative analysis of the periodontal health status and lung function status among patients with COPD, it was observed that the prevalence of periodontitis was significantly elevated in COPD patients [59]. Additionally, COPD patients with periodontitis exhibited poorer lung function compared to those without periodontitis [59]. Furthermore, upon evaluating the periodontal status in both pneumonia and non-pneumonia patients, it was found that individuals with periodontal diseases were susceptible to developing pneumonia in comparison to those without periodontal disease [60]. A meta-analysis conducted by Zeng et al. [61] demonstrated that patients with periodontal diseases had a significantly higher incidence of concurrent lung cancer, which illustrates that periodontal inflammation may affect respiratory epithelial cells and promote carcinogenesis.

Considering the critical roles played by *P. gingivalis* and *F. nucleatum* in the pathogenesis of periodontal diseases and relatively more literature reporting their involvement in respiratory infection, inflammation, and diseases, this review next focuses on the effects of both of two periodontal pathogenic bacteria on the development of respiratory diseases.

## 3. Specific Respiratory Diseases Relating to *P. gingivalis* and *F. nucleatum*

So far, a considerable amount of studies have accumulated to establish the links between *P. gingivalis*, *F. nucleatum*, and multiple respiratory diseases, including pneumonia, COPD, lung cancer, asthma, and so on (Table 1).

### 3.1. Pneumonia

It is widely acknowledged that dental plaque releases bacteria into saliva, which can potentially be inhaled into the lower respiratory tract, leading to the development of pneumonia [87]. Various types of oral anaerobic and facultative bacteria, such as *P. gingivalis* and *F. nucleatum*, have been isolated from infected lung fluid [88].

As a primary pathogen of chronic periodontitis [89], *P. gingivalis* has been cultured from the lung aspirates of approximately 40% of patients with aspiration pneumonia [63,90,91] and is similarly found in many lung infections leading to abscesses, necrotizing pneumonia, and pulmonary edema [92]. Aspiration pneumonia is considered a major health problem in the elderly [93,94] and poses a higher risk for patients with concomitant cognitive disorders such as Alzheimer’s disease [95]. Furthermore, although the underlying route of infection is not fully elucidated, the oropharyngeal tract is generally considered to be the most common route of infection in ventilator-associated pneumonia (VAP) [96]. Morillo et al. [64] detected the presence of *P. gingivalis* obtained in subglottic lavage samples from intubated and mechanically ventilated patients, suggesting a potential causal relationship between *P. gingivalis* and the development of VAP. A moderate amount of evidence shows that improved oral hygiene may reduce the risk of aspiration pneumonia in high-risk patients [97], which offers a potential perspective on preventing aspiration pneumonia.

The association between *F. nucleatum* and pneumonia has been suggested in recent studies. Hoffmeister et al. [78] reported a specific case of pneumonia where *F. nucleatum* infection was identified as the causative agent and inferred that the patient’s inadequate performed dental procedures were the likely source of the *F. nucleatum* infection. This finding implies that *F. nucleatum*, present in the oral cavity, may potentially migrate to the lungs and result in pneumonia. Moreover, in a study involving patients diagnosed with bacterial pneumonia, more than half (54.8%) of lower respiratory tract specimens tested positive for *F. nucleatum* by real-time PCR, further highlighting its potential involvement in pneumonia pathogenesis [76]. Moreover, recent studies have demonstrated the potential role of *F. nucleatum* to cause pneumonia in specific individuals. For patients with orotracheal intubation, *F. nucleatum* was found in their blood culture and demonstrated higher values in mini-bronchoalveolar lavage [77]. This finding demonstrates that *F. nucleatum* can be transmitted not only through the respiratory tract but also through the blood circulation to the lungs, potentially contributing to VAP [77]. What’s more, Bao et al. [80] reported that the pharyngeal *F. nucleatum* was significantly increased in COVID-19 patients and was higher in male than female patients, indicating that clinicians should pay careful attention to the potential *F. nucleatum* coinfection when treating COVID-19 patients. In addition, Wolff et al. [79] reported four cases of *F. nucleatum* bacteremia associated with coronavirus pneumonia. According to their study, pulmonary inflammation secondary to SARS-CoV-2 infection might promote the translocation of *F. nucleatum*, potentially leading to anaerobe bacteremia. Thus, by assessing the association between *F. nucleatum* and SARS-CoV-2, researchers may further understand the specific, synergistic, additive, or antagonistic actions between SARS-CoV-2 and other oral microorganisms, which may affect the outcomes of SARS -Cov-2 infection [98].

### 3.2. COPD

Studies have indicated that the lung microbiome samples are enriched with bacteria or bacterial products common to the oral cavity in patients with COPD [99,100]. Wu et al. [67] found that the abundance of *Porphyromonas* spp. was significantly higher in COPD than in non-COPD patients. And another study further specifically showed that the prevalence of *P. gingivalis* was higher in COPD patients than in the control participants [68]. Notably, *P. gingivalis* detected in tracheal aspirates from acute exacerbation of COPD (AECOPD) patients was highly homologous to the strains present in the corresponding dental plaque [66]. Moreover, the levels of *P. gingivalis* were higher in tracheal aspirates than in oral samples [66]. This evidence suggests that *P. gingivalis* colonizing the lower respiratory tract and lung is of oral origin and strongly associated with exacerbations of COPD. Additionally, patients with COPD showed a statistically significant negative correlation between forced expiratory volume in 1 s (FEV1%), the important index of lung function, and *P. gingivalis* content [68]. Thus, the presence of *P. gingivalis* may accelerate the decrease in lung function in COPD patients. Furthermore, Takahashi et al. [65] discovered that a normal IgG titer for *P. gingivalis*-related antibody can be used to predict frequent exacerbations. According to their study, patients with higher IgG titers had fewer exacerbations and a lower rate of frequent exacerbations than those with normal IgG titers [65], also suggesting the negative correlation between *P. gingivalis* and COPD.

Li et al. [81] conducted a study revealing a correlation between respiratory infections caused by *F. nucleatum* and the failure of antibiotic therapy as well as acute exacerbations of COPD. The researchers identified *F. nucleatum* in the tracheal aspirates of 60.8% of individuals experiencing AECOPD. Notably, similar to the observations made with *P. gingivalis*, the study found that as the quantity of *F. nucleatum* increased, patients’ FEV1% gradually declined [81]. These findings suggest a potential association among *F. nucleatum* infections, impaired response to antibiotic treatment, AECOPD, and a progressive decrease in lung function among affected individuals. Further research on the microbiological connection between periodontopathogens like *P. gingivalis* and *F. nucleatum* and COPD will make it possible to develop novel methods for identifying, monitoring, and treating COPD.

### 3.3. Lung Cancer

It has been shown that *P. gingivalis* may engage in the occurrence and development of multiple tumors, including lung cancer [101,102]. Perrone et al. [103] noted that the invasion of microorganisms to the respiratory tract as well as the lung can influence the pathogenesis, progression, and outcome of lung cancer. *P. gingivalis*-stained sections were significantly more frequent and intense in cancerous tissues of small cell lung cancer, lung adenocarcinoma, and lung squamous cell carcinoma, compared with adjacent lung tissues [69]. This may result from the microenvironment of lung cancer tissues, which is more favorable for *P. gingivalis* colonization and survival, thus causing an increase in the abundance of *P. gingivalis* [69]. Furthermore, lung cancer patients with *P. gingivalis* infection also had significantly lower survival rates and median survival times [69]. Moreover, through the assessment of IgG antibodies specific to *P. gingivalis* within the serum of lung cancer patients, a positive correlation has been established between the levels of these antibodies and the susceptibility to developing lung cancer when contrasted with a cohort of healthy controls [70,71]. This intriguing link between the level of antibodies targeting *P. gingivalis* and the associated risk of lung cancer highlights a potential role of *P. gingivalis* in lung cancer pathogenesis.

In addition, statistical analysis showed a correlation between the presence of *F. nucleatum* and the risk of developing systemic cancers, especially lung cancer [82]. In a manner akin to the observations made regarding *P. gingivalis*, there exists a positive correlation between serum levels of IgG antibodies targeting *F. nucleatum* and the occurrence of lung cancer [71]. This alignment between the antibody levels and lung cancer underscores a potential association between *F. nucleatum* and the pathogenesis of lung cancer, meriting further investigation. Chu et al. [83] found that airway-enriched *F. nucleatum* before anti-PD-1 treatment was associated with resistance to anti-PD-1 response in lung cancer, suggesting *F. nucleatum* may not only be related to the development of lung cancer, but also increase the resistance of cancer tissue to immunotherapy. While some investigations have documented the detection of *F. nucleatum* within pleural effusions of lung cancer patients [84], specific evidence establishing a direct correlation between *F. nucleatum* and lung cancer remains relatively limited. In broader terms, there exists a need for further extensive research to delve into the potential role of periodontopathogens in the intricate process of carcinogenesis.

### 3.4. Asthma

Examination of IgG antibody levels against *P. gingivalis* within serum samples has highlighted an observation—higher concentrations of IgG antibodies were significantly associated with a diminished prevalence of asthma [72]. Notably, a study involving an asthma mouse model, induced using ovalbumin, further substantiated this notion. The mice, sensitized with ovalbumin, were subsequently introduced to *P. gingivalis* subcutaneously. Interestingly, while *P. gingivalis* infection did not impact the inflammatory state of the mice, it did exhibit the ability to reduce airway responsiveness [73]. This intriguing finding provides support for a potentially negative correlation between *P. gingivalis* and the occurrence of asthma. This inverse association aligns harmoniously with the hypothesis suggesting that early infections could potentially act as preventive measures against allergic diseases [104].

### 3.5. Other Respiratory Diseases

The presence of *P. gingivalis* has been detected in the bronchoalveolar lavage fluid (BALF) of certain emphysema patients who have undergone lung transplantation [74]. Furthermore, a documented case highlighted a subcutaneous chest wall abscess attributed to *P. gingivalis*, as reported by Akane et al. [75]. An examination of sputum from cystic fibrosis patients utilizing 16S rRNA gene sequencing has illuminated a substantial colonization of *F. nucleatum* within the airways, with a relative abundance exceeding 50% [86]. Furthermore, an investigation into the levels of IgA antibodies against *F. nucleatum* within sputum from patients experiencing acute exacerbations of chronic bronchitis revealed a noteworthy finding. The antibody levels were, on average, 3.5 times higher in patients with acute exacerbations in comparison to healthy controls [85]. These cumulative observations shed light on the multifaceted interactions between *P. gingivalis* and *F. nucleatum* and respiratory health.

## 4. Potential Pathogenic Mechanisms of *P. gingivalis* and *F. nucleatum* in Respiratory Diseases

### 4.1. Induction of Mucus Hypersecretion and Airway Inflammation

Mucus hypersecretion and persistent airway inflammation are common pathological features of several respiratory diseases, such as pneumonia [105], COPD [106], and asthma [107]. In healthy individuals, airway mucus acts as an extracellular barrier to protect the tract from physical, chemical, and biological irritants [108]. However, in the case of lung infection, the large amount of mucus secreted from the airways and lungs can seriously affect the ventilation function of the airways and the exchange of oxygen in the alveoli, leading to hypoxemia and even asphyxia, which is one of the important causes of death in severe respiratory diseases [109]. Moderate inflammation of the organism can clear bacteria and protect the lower respiratory tract and lung in the face of bacterial invasion, but long-term chronic inflammation can cause irreversible damage to the organism [110]. Excessive inflammatory responses have even been linked to the development of cancer [111].

**Figure 1:**
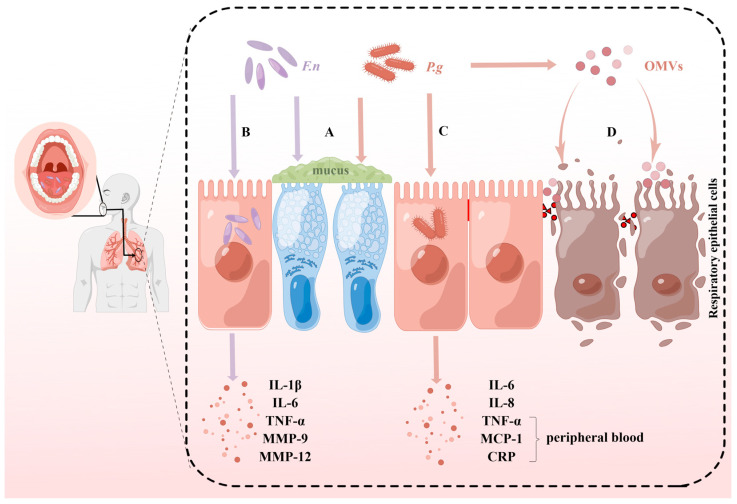
Separate pathogenic effects of *P. gingivalis* and *F. nucleatum.* (**A**) Both *P. gingivalis* (*P.g*) and *F. nucleatum* (*F.n*) can promote the expression of MU5AC genes and proteins thereby causing increased mucus in the lungs. (**B**) *F. nucleatum* invades the lung and induces the production of IL-1β, IL-6, TNF-α, MMP-9, and MMP-12, leading to lung inflammation and degradation of the extracellular matrix and basement membrane of the airways and lungs. (**C**) *P. gingivalis* invades the lung and induces the production of IL-6, IL-8, TNF-α, MCP-1, and CRP, leading to lung inflammation. (**D**) Outer membrane vesicles (OMVs) secreted by *P. gingivalis* containing a variety of virulence factors, such as LPS, fimbriae, and C-terminal domain (CTD) proteins, on the one hand, cause changes in cell morphology and promote apoptosis. On the other hand, OMVs can disrupt the tight junctions between cells and destroy the respiratory barrier.

MUC5AC is a mucus-forming mucin widely found in the human respiratory tract [112]. Both *F. nucleatum* and *P. gingivalis* enhance the expression of the MUC5AC gene and protein in the lungs of mice [113,114] (Figure 1A), and the increased concentration of MUC5AC contributes to the development and exacerbation of COPD [115].

Heat-inactivated *P. gingivalis* can induce IL-6 and IL-8 mRNA expression in human bronchial epithelial cells and pharyngeal epithelial cells via toll-like receptor 2 (TLR-2), thereby promoting the production of proinflammatory cytokines [9] (Figure 1C). Similar results have been observed in animal models. TNF-α, IL-6, monocyte chemoattractant protein-1 (MCP-1), and C-reactive protein (CRP) levels in peripheral blood were dramatically increased in mice 24 h after injection of *P. gingivalis* into the trachea [116] (Figure 1C). IL-6 is a pleiotropic cytokine that promotes T-helper 2 (Th2) cell- and Th17 cell-mediated immune responses. IL-8, a pro-inflammatory cytokine, promotes the production of extracellular matrix by lung fibroblasts, in addition to promoting the chemotaxis of neutrophils to the site of injury [117]. Elevated levels of IL-6 and IL-8 expression in sputum and plasma of COPD patients may be associated with severe acute exacerbations of COPD [118,119]. TNF-α is a potent NF-κB activator that amplifies neutrophil inflammation and activates macrophages, which may be associated with the development of COPD and lung cancer [120,121]. MCP-1 is an activator of monocytes, T-lymphocytes, and B-lymphocytes involved in the recruitment and activation of airway inflammatory cells [122]. CRP refers to a group of proteins that rise sharply in plasma when the body is exposed to infection or tissue damage and is a biomarker for assessing secondary infections in pneumonia and acute exacerbations of COPD [123,124,125]. It is worth noting that the production of these pro-inflammatory cytokines may be closely related to gingipain. Mice inoculated with *P. gingivalis* strains with knockout gingipain-related genes developed only transient and mild lung inflammation, without alterations in the levels of TNF-α, IL-6, and MCP-1, while all mice inoculated with wild-type *P. gingivalis* developed respiratory failure [116].

Similar to *P. gingivalis*, heat-inactivated *F. nucleatum* strongly induced IL-6 and IL-8 production in bronchial epithelial cells, pharyngeal epithelial cells, and alveolar epithelial cells in a density-dependent manner [126,127] (Figure 1B). And analogous results were found in another study of respiratory epithelial cells treated with *F. nucleatum* culture supernatants [128]. Li et al. [129] showed that *F. nucleatum* could upregulate the secretion of IL-1β, IL-6, and TNF-α in alveolar epithelial cells (Figure 1B). The mechanism by which *F. nucleatum* mediates the production of inflammatory mediators by multiple respiratory epithelial cells may be jointly dependent on heme secretion [127]. IL-1β is associated with macrophage activation and neutrophil inflammation. NOD-, LRR-, and pyrin domain-containing protein 3 (NLRP3) inflammasomes may induce an acute exacerbation of the respiratory inflammatory response by increasing IL-1β expression [130,131]. Matrix metalloproteinases (MMPs) can break down the extracellular matrix and basement membrane of the airways and lungs, thus participating in the reconstruction of the airways and lungs [132]. On top of that, MMPs regulate the activity of other proteases and cytokines [133]. Thus, MMPs play an essential role in the pathogenesis of respiratory diseases such as pneumonia [132] and COPD [134]. After the administration of *F. nucleatum* into the lung and trachea of mice, an increase in the expression of MMP-9 [135] and MMP-12 [136] was observed in the BALF of mice (Figure 1B). *F. nucleatum* induces MMPs expression potentially via activated mitogen-activated protein kinases (MAPKs) and NF-ĸB in human alveolar epithelial cells [135].

### 4.2. Cytotoxic Effects

As fundamental cellular processes, a balance must be maintained between autophagy and apoptosis to maintain basic cellular functions and tissue homeostasis [137]. OMVs of *P. gingivalis* induce cellular morphological changes such as cell shrinkage, membrane blebbing, and cytoplasmic expulsion in alveolar epithelial cells (Figure 1D), thereby reducing cellular activity and promoting apoptosis in a dose- and time-dependent manner [138,139]. Additionally, OMVs produced by *P. gingivalis* can also disrupt the intact distributions of tight junctions between cells [138] (Figure 1D), which may cause damage to the respiratory barrier, thereby leading to pneumonia, COPD, and other lung diseases [140,141,142]. OMVs are bilayer spherical vesicles formed and secreted by *P. gingivalis* and contain a variety of virulence factors of *P. gingivalis*, such as LPS [143], fimbriae [144], and C-terminal domain (CTD) proteins [145]. OMVs protect proteins from hydrolysis and transport to other parts of the body, and also act synergistically with other bacterial products [30]. Therefore, *P. gingivalis* can still exert toxic effects via OMVs even in areas that *P. gingivalis* cannot reach. Elevated perforin was detected in the BALF of mice inoculated with *F. nucleatum* [136]. Natural killer (NK) cells can kill autologous lung epithelial cells via the perforin–granzyme pathway [146].

### 4.3. Synergistic Pathogenic Effects with Respiratory Pathogens

Since both *P. gingivalis* and *F. nucleatum* are obligate anaerobic bacteria, they have difficulty surviving in the aerobic environment of the lungs for long periods. Neither *P. gingivalis* nor *F. nucleatum* infection alone can cause significant lung disease in mice [129,147]. However, *P. gingivalis* and *F. nucleatum* may interact with other respiratory pathogens to promote the development of lung diseases.

#### 4.3.1. *P. gingivalis* and *Streptococcus pneumoniae*

**Figure 2:**
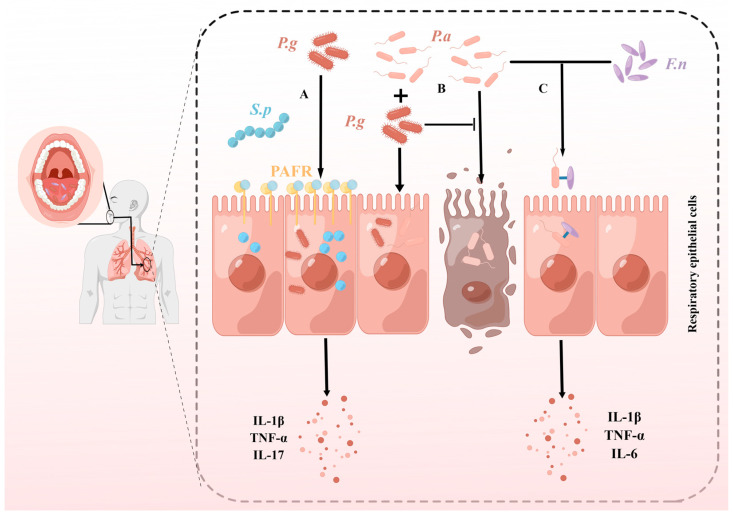
Synergistic pathogenic effects of *P. gingivalis* and *F. nucleatum* with respiratory pathogens. (**A**) *P. gingivalis* (*P.g*) induced the expression of platelet-activating factor receptor (PAFR), which promoted the invasion of respiratory epithelial cells by *Streptococcus pneumoniae* (*S.p*). Mixed infection with both bacteria significantly increased the levels of IL-1β, TNF-α, and IL-17 in the lungs, compared with *S. pneumoniae* infection alone. (**B**) *P. gingivalis* and *P. aeruginosa* (*P.a*) co-cultures can mutually invade respiratory epithelial cells, although *P. gingivalis* inhibited *P. aeruginosa*-induced apoptosis via the STAT3 signaling pathway during the early stage of co-infection. (**C**) *F. nucleatum* (*F.n*) and *P. aeruginosa* co-aggregate to form more complex biofilms and jointly invade respiratory epithelial cells. Co-infection of the two species effectively increased the levels of IL-1β, TNF-α, and IL-6 in the lungs.

*P. gingivalis* culture supernatant promotes *S. pneumoniae* adhesion to cells by inducing increased platelet-activating factor receptor (PAFR) expression in alveolar epithelial cells [148] (Figure 2A). Moreover, among the main components of *P. gingivalis* culture supernatant (LPS, fimbriae, and gingipain), only Arg-gingipain enhanced the adhesion of *S. pneumoniae* [148]. PAFR is a transmembrane G protein-coupled receptor that not only binds to PAF, and thus mediates inflammation [149], but also a primary receptor for *S. pneumoniae* adhesion and invasion of the lung [150]. Increased PAFR expression in respiratory epithelial cells has been proven to be associated with a variety of lung diseases such as pneumonia [150], COPD [151], and lung cancer [152]. Mixed infection with *P. gingivalis* and *S. pneumoniae* enhanced gene as well as protein expression of pro-inflammatory cytokines and chemokines such as IL-1β, TNF-α, and IL-17 in mouse lungs compared with *S. pneumoniae* infection alone [147] (Figure 2A). In contrast, *P. gingivalis* culture supernatant infection alone did not induce an inflammatory response in the lungs of mice [147], so *P. gingivalis* may play an indirect role in pneumonia by enhancing the inflammation caused by *S. pneumoniae*.

#### 4.3.2. *P. gingivalis* and *Pseudomonas aeruginosa*

*P. gingivalis* and *P. aeruginosa* co-cultures can mutually invade alveolar epithelial cells, but, interestingly, *P. gingivalis* inhibits *P. aeruginosa*-induced apoptosis in the early stages of infection [153] (Figure 2B). *P. gingivalis* may help *P. aeruginosa* cause long-term chronic infection in the lung by delaying the apoptosis of host cells. The signal transducer and activator of transcription 3 (STAT3), a DNA-binding protein, is involved in the regulation of various cellular processes such as proliferation, survival, differentiation, and angiogenesis [154]. *P. gingivalis* regulates *P. aeruginosa*-induced apoptosis through the STAT3 signaling pathway [153].

#### 4.3.3. *F. nucleatum* and *P. aeruginosa*

*F. nucleatum* can promote the proliferation of *P. aeruginosa* and co-form with it a more structurally and functionally diverse biofilm [81] (Figure 2C). When *F. nucleatum* and *P. aeruginosa* were co-cultured, *F. nucleatum* could survive and reproduce under aerobic conditions [81]. This may be because *P. aeruginosa* growth consumes dissolved oxygen in the medium, thus creating anaerobic conditions for *F. nucleatum* [155]. When *F. nucleatum* and *P. aeruginosa* co-infected with alveolar epithelial cells, the two bacteria could adhere to the alveolar epithelial cells after co-aggregation and also enhanced the invasive ability of both bacteria [129] (Figure 2C). *F. nucleatum* increased *P. aeruginosa*-induced secretion of IL-1β, TNF-α, and IL-6 in pulmonary epithelial cells and also amplified the cytotoxic effects of *P. aeruginosa* on pulmonary epithelial cells [129] (Figure 2C). The coexistence of *F. nucleatum* and *P. aeruginosa* appears to exacerbate the detrimental impact on the lungs of individuals with COPD. This observation potentially elucidates why patients who are co-infected with both *F. nucleatum* and *P. aeruginosa* experience a more accelerated decline in lung function [81]. Furthermore, it has been observed that as the quantity of *F. nucleatum* increases, the decrease in lung function becomes more pronounced [81]. These findings suggest that the presence of *F. nucleatum* amplifies the deleterious effects caused by *P. aeruginosa*, contributing to a more rapid deterioration of lung function in COPD patients. In dual-species biofilms, the characteristic adhesin FadA of *F. nucleatum* can reduce the antibiotic sensitivity of biofilms [81], which may be one of the underlying factors contributing to the increased risk of exacerbations and poorer outcomes in COPD patients with *P. aeruginosa* co-infection. [156].

## 5. Discussion and Perspectives

In vitro cellular experiments and animal studies together provide ample evidence that *P. gingivalis* and *F. nucleatum* adversely affect respiratory diseases through a variety of possible mechanisms. *P. gingivalis* and *F. nucleatum* and their products may reach the lower respiratory tract and lung through saliva aspiration and blood circulation, causing chronic inflammation and apoptosis in the respiratory system. On the one hand, interacting with other respiratory pathogens exerts stronger virulence on the lungs. *P. gingivalis* and *F. nucleatum* can hardly survive in the lungs alone for a long time in an aerobic environment but may colonize the lungs in synergy with other respiratory pathogenic bacteria such as *P. aeruginosa*. In turn, colonized *P. gingivalis* and *F. nucleatum* can enhance the virulence of respiratory pathogens (including the induction of apoptosis in inflammation). Chronic, low-grade, unresolved inflammation underlies the development of multiple diseases throughout the body including respiratory and periodontal diseases [157,158]. The potential function of *P. gingivalis* and *F. nucleatum* links periodontal diseases and respiratory diseases. More studies are still needed to clarify the mechanisms of the role of *P. gingivalis* and *F. nucleatum* in the pathogenesis of respiratory diseases. Considering the critical role played by *P. gingivalis* and *F. nucleatum* in the pathogenesis of periodontal diseases, future research could focus on the link between periodontal diseases and respiratory diseases by specific periodontal pathogenic bacteria.

More importantly, the achievements of basic research should be translated into clinical applications, providing a theoretical basis for the development of new treatment options for periodontal and respiratory diseases. Although there are randomized controlled trials demonstrating that periodontal treatment improves lung function and reduces the frequency of pulmonary disease exacerbations [159,160,161,162,163,164], there is no clear evidence that the treatment against *P. gingivalis* and *F. nucleatum* reduces the risk or incidence of respiratory diseases. Gingipains and FadA are, respectively, the main virulence factors of *P. gingivalis* and *F. nucleatum* that induce the production of pro-inflammatory cytokines in the lower respiratory tract and lung and act synergistically with other respiratory pathogenic bacteria [165,166]. Thus, targeted therapy against key virulence factors produced by *P. gingivalis* and *F. nucleatum*, such as gingipains and FadA, may ameliorate respiratory diseases.

## Figures and Tables

**Table 1:** *P. gingivalis* and *F. nucleatum* correlate with respiratory diseases.

Periodontopathogen	Respiratory Disease	Correlation	References	Year
*Porphyromonas gingivalis*	Pneumonia	*P. gingivalis* was cultured from the lung aspirates of patients with aspiration pneumonia.	[62,63]	1974, 2005
		*P. gingivalis* was detected in subglottic lavage samples from intubated and mechanically ventilated patients.	[64]	2021
	COPD	Patients with higher IgG titers for *P. gingivalis*-related antibodies had fewer exacerbations and a lower rate of frequent exacerbations than those with normal IgG titers.	[65]	2012
		1. *P. gingivalis* detected in tracheal aspirates from AECOPD patients was highly homologous to the strains present in the corresponding dental plaque. 2. The levels of *P. gingivalis* were higher in tracheal aspirates than in oral samples from AECOPD patients.	[66]	2014
		The abundance of *P. gingivalis* was significantly higher in COPD than in non-COPD patients.	[67]	2017
		Patients with COPD showed a statistically remarkable negative correlation between FEV1% and *P. gingivalis* content.	[68]	2019
	Lung Cancer	1. *P. gingivalis*-stained sections were significantly more frequent and intense in cancerous tissues of small cell lung cancer, lung adenocarcinoma, and lung squamous cell carcinoma, compared with adjacent lung tissues. 2. Lung cancer patients with *P. gingivalis* infection also had significantly lower survival rates and median survival times.	[69]	2021
		The risk of developing lung cancer shows a positive correlation with the serum levels of IgG antibodies directed against *P. gingivalis*.	[70,71]	2023
	Asthma	Higher IgG concentrations of *P. gingivalis*-related antibodies were significantly associated with a diminished prevalence of asthma.	[72]	2006
		Subcutaneously injected *P. gingivalis* reduces airway responsiveness in ovalbumin-induced asthma mice.	[73]	2010
	Other Respiratory Diseases	The presence of *P. gingivalis* has been detected in the BALF of certain emphysema patients.	[74]	2011
		A case of subcutaneous chest wall abscess caused by *P. gingivalis* infection is reported.	[75]	2022
*Fusobacterium nucleatum*	Pneumonia	A total of 54.8% of lower respiratory tract specimens of patients with bacterial pneumonia tested positive for *F. nucleatum* by real-time PCR.	[76]	2017
		*F. nucleatum* was found in the blood culture of patients with orotracheal intubation and demonstrated higher values in mini-bronchoalveolar lavage, potentially contributing to VAP.	[77]	2018
		Reports the case of a patient who suffered from pneumonia with chest wall invasion by *F. nucleatum*.	[78]	2021
		Reports four cases of *F. nucleatum* bacteremia associated with coronavirus pneumonia.	[79]	2021
		The pharyngeal *F. nucleatum* was significantly increased in COVID-19 patients and was higher in male than female patients.	[80]	2021
	COPD	1. A total of 60.8% of individuals with AECOPD had *F. nucleatum* present in their tracheal aspirates. 2. FEV1% of AECOPD patients gradually decreased as the number of *F. nucleatum* rose.	[81]	2020
	Lung Cancer	The presence of *F. nucleatum* is associated with the risk of developing systemic cancers, especially lung cancer among postmenopausal females.	[82]	2016
		Airway-enriched *F. nucleatum* before anti-PD-1 treatment was associated with resistance to anti-PD-1 response in lung cancer.	[83]	2022
		*F. nucleatum* was detected within pleural effusions of lung cancer patients.	[84]	2023
		The risk of developing lung cancer shows a positive correlation with the serum levels of IgG antibodies directed against *F. nucleatum*.	[71]	2023
	Other Respiratory Diseases	IgA antibody levels against *F. nucleatum* in the sputum of patients with acute exacerbations of chronic bronchitis were on average 3.5 times higher than in healthy controls.	[85]	2003
		Examination of sputum from cystic fibrosis patients utilizing 16S rRNA gene sequencing illuminated that the relative abundance of *F. nucleatum* exceeded 50%.	[86]	2015

COPD, chronic obstructive pulmonary disease; AECOPD, acute exacerbation of COPD; FEV1%, forced expiratory volume in 1 s; BALF, bronchoalveolar lavage fluid; VAP, ventilator-associated pneumonia.
